# Current understanding of CTLA-4: from mechanism to autoimmune diseases

**DOI:** 10.3389/fimmu.2023.1198365

**Published:** 2023-07-11

**Authors:** Md Munnaf Hossen, Yanmei Ma, Zhihua Yin, Yuhao Xia, Jing Du, Jim Yi Huang, Jennifer Jin Huang, Linghua Zou, Zhizhong Ye, Zhong Huang

**Affiliations:** ^1^ Shenzhen Futian Hospital for Rheumatic Diseases, Shenzhen, China; ^2^ Department of Immunology, Biological Therapy Institute, Guangdong Provincial Key Laboratory of Regional Immunity and Diseases, Health Science Center, Shenzhen University, Shenzhen, China; ^3^ Joint Research Laboratory for Rheumatology of Shenzhen University Health Science Center and Shenzhen Futian Hospital for Rheumatic Diseases, Shenzhen, China; ^4^ Department of Laboratory Medicine, Peking University Shenzhen Hospital, Shenzhen, China; ^5^ Department of Psychology, University of Oklahoma, Norman, OK, United States; ^6^ Department of Chemistry and Biochemistry, University of Oklahoma, Norman, OK, United States; ^7^ Department of Rehabilitation Shenzhen Futian Hospital for Rheumatic Diseases, Shenzhen, China

**Keywords:** CTLA-4, autoimmunity, regulatory T cell, autoimmune disease, immune regulation

## Abstract

Autoimmune diseases (ADs) are characterized by the production of autoreactive lymphocytes, immune responses to self-antigens, and inflammation in related tissues and organs. Cytotoxic T-lymphocyte antigen 4 (CTLA-4) is majorly expressed in activated T cells and works as a critical regulator in the inflammatory response. In this review, we first describe the structure, expression, and how the signaling pathways of CTLA-4 participate in reducing effector T-cell activity and enhancing the immunomodulatory ability of regulatory T (Treg) cells to reduce immune response, maintain immune homeostasis, and maintain autoimmune silence. We then focused on the correlation between CTLA-4 and different ADs and how this molecule regulates the immune activity of the diseases and inhibits the onset, progression, and pathology of various ADs. Finally, we summarized the current progress of CTLA-4 as a therapeutic target for various ADs.

## 1 Introduction

**Figure 1:**
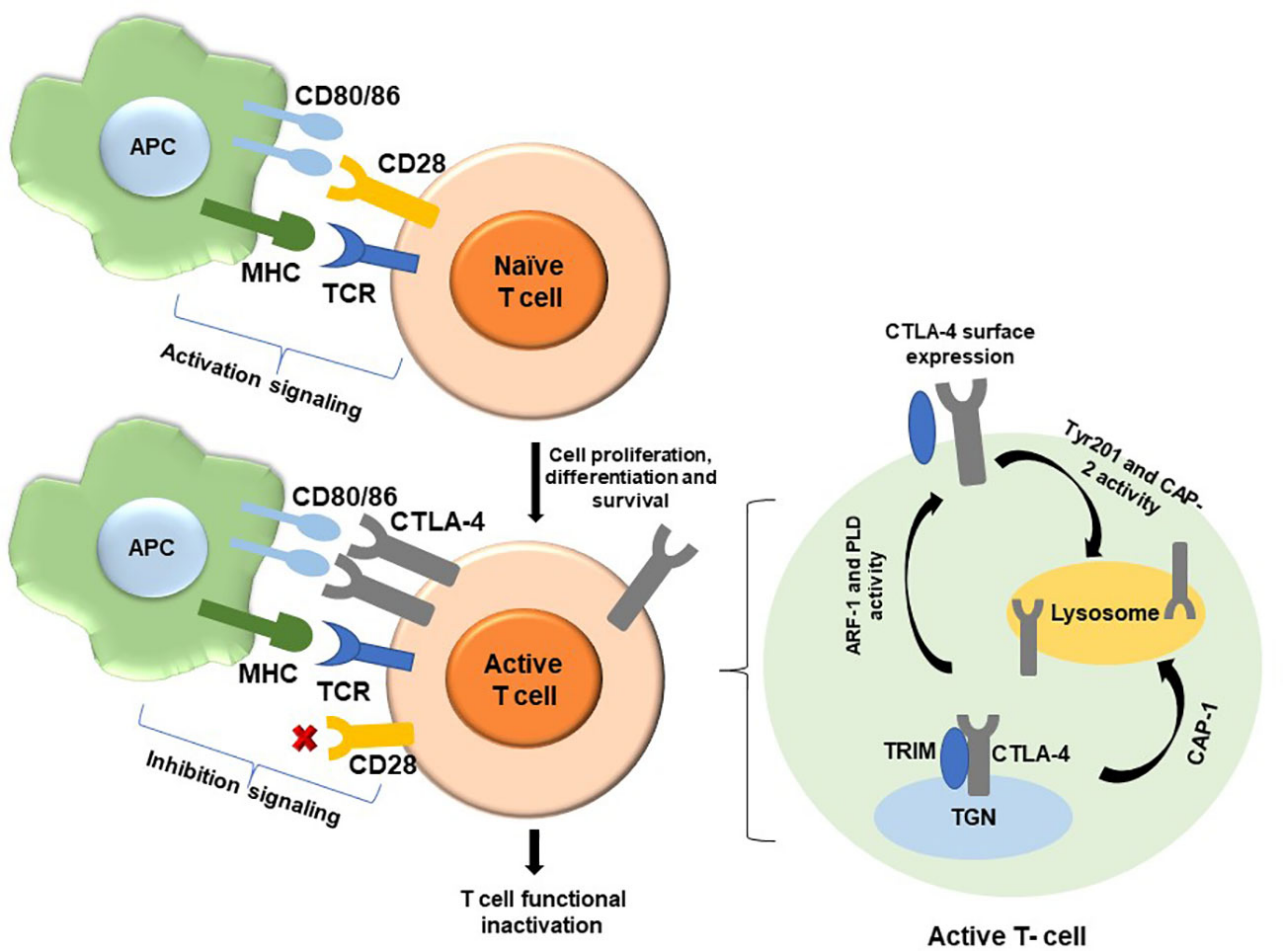
Signaling pathway of T-cell activation and CTLA-4 surface expression. Two signals are essential for T-cell activation. One is TCR-MHC signaling and another one is CD28-CD80/86 co-stimulation. After successful co-stimulation, naïve T cells become active and perform their function. After activation, T cells expresses CTLA-4 and CTLA-4 binds with CD80/86 molecules and functionally inactivated T cell (left panel). The newly synthesized CTLA-4 binds with TRIM in the TGN and comes out on the cell surface. PLD and ARF-1 are also responsible for externalization of CTLA-4. On the other side, CTLA-4 binds with CAP-1 and directs transport to lysosomal compartment. From the surface, dephosphorylated tyrosine 201 of CTLA-4 binds with clathrin adaptor protein-2 (CAP-2) and transports endosome to lysosome (right panel). APC, Antigen-presenting cell; TCR, T-cell receptor; MHC, Major histocompatibility complex; CTLA-4, Cytotoxic T lymphocyte antigen 4; CD28, TRIM, Transmembrane adaptor T-cell receptor interacting molecule; TGN, Trans Golgi network; PLD, Phospholipase D; ARF-1, GTPase ADP ribolysation factor-1; CAP-1, Clathrin adaptor protein-1; CAP-2, Clathrin adaptor protein-2.

CTLA-4 is a T-cell co-receptor, also known as CD152. Compared to CD28, CTLA-4 has a superior binding affinity with B7 family molecules, including CD80 and CD86 on antigen-presenting cells (APCs). Although CTLA-4 binds with B7 co-stimulatory receptors, it plays a negative role in the activation of T cells (1). Following the T-cell receptor (TCR) recognizing the antigen presented by the major histocompatibility complex (MHC) of APC, the CD28 of T cells binds to B7 of APC, which initiates a signaling cascade and leads to T-cell activation. CTLA-4s express and move to the cell membranes after T-cell activation, take over B7 from CD28, and suppress T-cell activity (2, 3). The coordination of CTLA-4 and CD28 maintains the balance of T-cell immunity in the body, especially after infection and the onset and progression of autoimmune disease. However, the precise immune-regulating mechanism of CTLA-4 in T cells is debatable (4, 5) (**Figure 1**).

In recent years, CTLA-4 has been shown to play a crucial role in immune checkpoint-based therapeutics, especially in cancer treatment, by using monoclonal antibodies against the molecule (6). Moreover, this regulatory molecule has been intimately involved in the treatment of autoimmune diseases (7). Elucidating the immunoregulatory mechanisms and roles of CTLA-4 in autoimmune diseases will provide potent immunotherapy targets for these diseases. In this review, we focused on the immunomodulatory role of CTLA-4 in T-cell immunity, discussed the key molecular signaling pathways mediated by CTLA-4, and summarized the latest immunoregulatory effects of CTLA-4 in various autoimmune diseases, especially its role in the progress and pathogenesis of the diseases and its clinical application in the diseases.

## 2 Gene and expression mechanism of CTLA-4

In humans and mice, the *CTLA-4* gene consists of 4 exons encoded by chromosomes 2 and 1, respectively. Exon 1 provides the sequence for the leading peptide, while exon 2 has a CD80/CD86 binding site and a dimerization site. Exon 3 comprises a transmembrane region, while exon 4 contains the cytoplasmic tail (8). The CTLA-4 gene has distinct isoforms in humans and mice. In humans, there is a full-length CTLA-4 mRNA (flCTLA-4, containing exons 1 to 4) and a soluble cytotoxic T-lymphocyte antigen 4 (sCTLA-4) that is detectable in serum; however, it does not contain exon 3 (9, 10). The half-life of flCTLA-4 mRNA is longer than that of sCTLA-4 mRNA (11). In murine T cells, an extra CTLA-4 transcript dubbed ligand-independent CTLA-4 (liCTLA-4) is produced, which contains exons 1, 3, and 4 (12, 13).

*In vitro* studies reported that inhibiting NF-AT activity in T cells significantly reduced CTLA-4 transcription, suggesting that the activity of NF-AT was positively correlated with protein expression (14, 15). Upregulated CTLA-4 expression was also regulated by cyclic AMP (cAMP) (16). The mRNA of CTLA-4 was detected in T cells after 1 h of T-cell receptor (TCR) ligation and reached a peak approximately 24–36 h after T cells were activated by antigen (17, 18).

The level of CTLA-4 is closely regulated by numerous factors, such as ligand-inducing expression, cell surface translocation, fast internalization, recycling, and degradation. CTLA-4 is induced by TCR ligation and forms a complex with the T-cell receptor interacting molecule (TRIM) in the trans-Golgi network (TGN), which promotes protein transfer into the cell surface (19). Externalization of CTLA-4 is also facilitated by guanosine triphosphatases (GTPases), adenosine diphosphate ribosylation factor-1 (ARF-1), phospholipase D (PLD), calcium influx (20), and Rab11 (21). On the other hand, CTLA-4 internalization is regulated by both clathrin-dependent and clathrin-independent pathways. For the dependent pathway, the cell surface CTLA-4 associates with clathrin adaptor protein-1 (CAP-1) and clathrin adaptor protein-2 (CAP-2); for the independent pathway, the CTLA-4 binds to dynamin. After internalization, CTLA-4 is either delivered into lysosomes or endosomes (22, 23). To maintain the intracellular stable state of CTLA-4, it binds with CAP-1 in the TGN of T cells and is then transported to the lysosomal compartments for degradation (21). Phosphorylation of CTLA-4 by Lck and Fyn tyrosine kinases inhibits this interaction and blocks the trafficking of CTLA-4, thus prolonging the retention of the protein on the cell membrane and reducing degradation of the protein (24) (**Figure 1**).

## 3 The main immunoregulatory mechanism of CTLA-4

### 3.1 CTLA-4 with CD28 competing B7 on activated antigen-presenting cells

**Figure 2:**
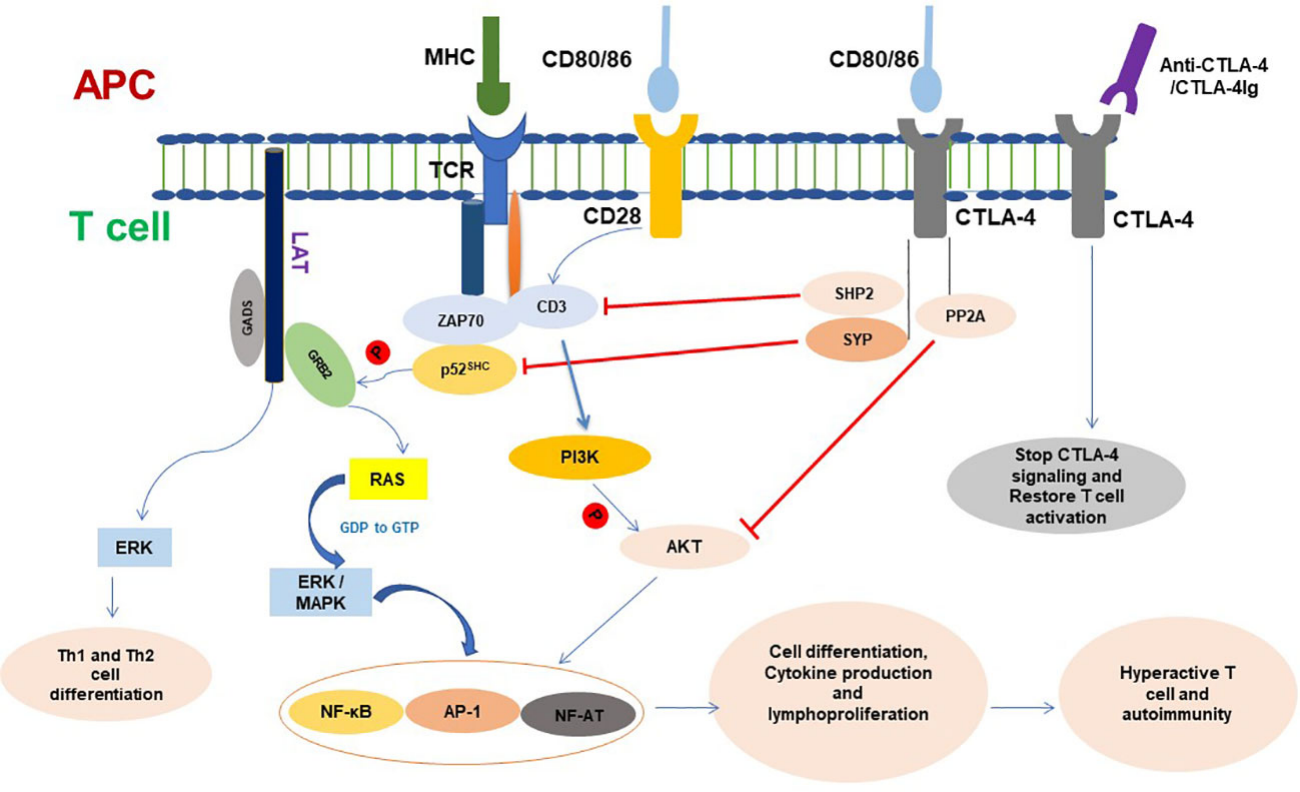
CTLA-4 signaling in T cell and functioning. CTLA-4 targets different molecules to inhibit T- cell activation and functioning. Once conjugated, T cell with APC initiates different signaling and T-cell proliferation and produces cytokines. CTLA-4 is expressed in cell surface of active T cells and binds with CD80/86. CTLA-4 signaling dephosphorylates TCR signaling and inhibits CD3 and ZAP70 signaling molecules and ultimately inhibits phosphorylation of GRB2 to RAS pathway. CTLA-4 inhibits Akt phosphorylation and activation and plays a negative role on the regulation of cell cycle and inhibits the transcription factors nuclear factor κB (NF-κB), AP-1, and NF-AT activation. Ligation of CTLA-4-CTLA-4Ig resumes T-cell signaling and functioning. APC, Antigen-presenting cell; AP-1, Activator protein 1; TCR, T-cell receptor; MHC, Major histocompatibility complex; CTLA-4, Cytotoxic T lymphocyte antigen 4; SYP, Tyrosine phosphatase synaptophysin; GRB2, Growth Factor Receptor-Bound 2; ZAP70, Zeta-chain associated protein kinase 70; NF-AF, Nuclear factor of activated T cells; ERK, Extracellular signal-regulated kinase; MAPK, Mitogen-activated protein kinase; PI3K, Phosphatidylinositol 3-kinase; PP2A, Protein phosphatase 2A.

The activation of T cells depends on the TCR binding to a specific antigen presented by the MHC of APCs. However, this recognition, the first T-cell activation signal, is not sufficient to cause T-cell activation (25, 26). To fully activate T cells, a second activation signal called co-stimulation is required. This signal is provided by a T cell’s inducible CD28 receptor, which binds to its ligand CD80 or CD86 on activated professional APCs (26). The activation of CD28 stimulates glucose absorption and cell cycle progression in T cells by increasing the expression of the anti-apoptotic proteins Bcl-X (Bcl-xL) and interleukin-2 (IL-2) to reduce apoptosis and increase proliferation of T cells (27–29). Without CD28 signaling, T cells will enter clonal anergy and apoptosis (30). When T cells receive the first and second activation signals, the cells become completely activated, and antigen-specific effector cells expand in peripheral lymphoid organs (31). If the antigen of the pathogen causes an expansion of particular T cells, they will assault contaminated cells and tissue at the site of infection. If the proliferated cells are due to self-antigen, they will move to target tissue and organs, cause inflammation, and damage the self-cell, tissue, and organ (**Figure 2**).

CTLA-4 is expressed in activated T lymphocytes and is transported to the cell membrane. Despite the fact that CD80 and CD86 are ligands found on APCs that CTLA-4 and CD28 both share, CTLA-4 has a considerably higher affinity for these ligands than CD28; therefore, it preferentially binds to CD80/CD86 and transmits inhibitory signals to prevent CD28-mediated T-cell activation (1). On the cell membrane, CTLA-4 is phosphorylated by Fyn and Lck. The phosphorylated CTLA-4 recruits SHIP2, which dephosphorylates CD3 and linkers for activation of T cells (LAT). Thus, CTLA-4s disrupt TCR/CD3 and CD28/B7 signaling and result in downregulating T cells’ activity, immune response, and inflammatory cytokine production (32, 33), cell cycle progression, and the transcription factors nuclear factor B (NF-κB), activator protein 1 (AP-1), and NF-AT (34, 35) **(** **Figure 2**).

In regulatory T (Treg) cells, CTLA-4 is constitutively expressed and required for the cell’s immune suppressive activity (36). It interacts with CD80/CD86 on dendritic cells (DCs), which transmit the inhibitory signal to the APCs, downregulating the expression of CD80/CD86 and upregulating the expression of indoleamine-2,3-dioxygenase (IDO) in DCs, thus endowing DCs with immune tolerance properties (37). IDO released from DCs acts as an immune regulator for T cells by depleting tryptophan (38, 39). Deficiency of CTLA-4 in Treg cells impairs the immune suppressive function of the cells *in vivo* (40) and leads to abnormal activation and expansion of conventional T cells (41). On the other hand, CTLA-4 has been found to promote the generation of CD4^+^CD25^+^ regulatory T cells by increasing FoxP3 expression that is induced by transforming growth factor (TGF-β) (42). So far, the CTLA-4-mediated signaling in T cells and immune response in DCs still have not yet been completely elucidated; further study will not only help to explain T cells’ and DCs’ immune regulation but also help to understand the mechanism for other immune cell regulation.

### 3.2 CTLA-4/SYP/p52^SHC^ pathway

The activation of TCR is initiated by binding the antigen presented by the MHC of DCs, which causes tyrosine phosphorylation of CD3. The adapter molecule p52^SHC^ is recruited by activated CD3 and tyrosine phosphorylated by Lck. The phosphorylated p52^SHC^ associates with SH2 and SH3 domains containing protein growth factor receptor bound protein (GRB2), and via the GRB2, with the guanine nucleotide exchange factor Son-of-Sevenless (SOS) and Ras form a complex (CD3/p52^SHC^/GRB2/SOS/Ras), which regulates the activity of the Ras signal (43, 44). It has been demonstrated that the tyrosine phosphatase synaptophysin (SYP) binds to the tyrosine phosphorylated YVKM motif in the cytoplasmic tail of CTLA-4. The CTLA-4-associated SYP exhibits tyrosine phosphatase activity towards p52^SHC^. The dephosphorylation of p52^SHC^ interferes with the binding affinity of p52^SHC^ to GRB2, thus disrupting the p52^SHC^/GRB2/SOS/Ras complex and resulting in Ras being unable to exchange GDP with GTP. This indicates that the regulatory effect of CTLA-4 on TCR-Ras signaling is via dephosphorylation of p52^SHC^ by SYP (45) (**Figure 2**).

P52^SHC^ has also been shown to be a component in C-X-C chemokine receptor type 4 (CXCR4) signaling. The binding of stromal-derived-factor-1α (SDF-1α) to CXCR4 results in activation of the receptor and association with Lck, which promotes phosphorylation of CD3 and p52^SHC^ and assembly of the p52^SHC^/ZAP-70/Vav complex, implying that the TCR transactive pathway can be triggered by CXCR4 (46). On the other hand, given the tyrosine phosphatase SYP for p52^SHC^ associated with CTLA-4, the complex of SYP/CTLA-4 may play a role in disrupting CXCR4-mediated chemotaxis and the activity of TCR signaling.

### 3.3 CTLA-4/PI3K/AKT pathway

Phosphatidylinositol (3,4,5)-trisphosphates (PIP3), the product of PI3K, recruits the pleckstrin homology (PH) domain protein AKT to the membrane. The activation of AKT occurs through the phosphorylation of Thr-308 by PH domain kinase 1 (PDK1) and Ser-473 via mechanistic target of rapamycin complex 2 (mTORC2) (47, 48). The activated AKT is involved in many cellular functions such as immune regulation, cell proliferation, metabolism, survival, and anti-apoptosis by mediating the transcriptional factor activities of NF-κB, NFAT, and AP-1 (35, 49).

As early as 1995, PI3 kinase (PI3K) was reported to bind to the cytoplasmic pYVKM motif of CTLA-4, but the role of CTLA-4 in the PI3K/AKT pathway is still not fully understood (50, 51). The activation of PI3K by CTLA-4 signaling was demonstrated by its ligand stimulation increasing the phosphorylation of AKT (52). The function of CTLA-4 is closely related to that of its partners, serine/threonine phosphatase PP2A (PP2A) and tyrosine phosphatase SHIP2 (SHIP2). Phosphorylated CTLA-4 has been demonstrated to be associated with PP2A in its cytoplasmic tail (53) and SHIP2 in its pYVKM motif (54), but another study showed that CTLA-4 interaction with SHIP2 is indirect, possibly via PI3K (55). Differences with Schneider et al.’s study and Parry et al.’s study showed that CTLA-4 inhibited AKT activity through its partner PP2A, which was confirmed by the PP2A inhibitor okadaic acid or deleting its lysine-rich domain KLESS (a motif required for PP2A binding in CTLA-4) (56). This implies that the association of PP2A and its phosphatase activity is necessary for CTLA-4 to inhibit the activity of AKT. Interestingly, this inhibition preserved PI3K activity, which was further demonstrated by the PI3K inhibitor LY294002 reducing CTLA-4-induced Bcl-xL production (56). The function of SHIP2 in CTLA-4 signaling has not yet been elucidated, as their interaction pattern is still under debate, and SHIP2 has been shown to increase ERK activity (57). However, it is still possible for SHIP2 to dephosphorylate some TCR signaling proteins and reduce TCR-mediated T-cell activation (58, 59). On the other hand, Wu et al. showed that SHIP2 is required for PI3K/AKT activation via EGF, PDGF, and IGF signaling (60). AKT phosphorylates the apoptotic protein BL2 associated agonist of cell death (BAD), which releases the anti-apoptosis protein B-cell lymphoma 2 (BcL-2) or BcL-XL from the heterodimer of BAD-BcL-2 and BAD-BcL-XL (61).

Studies have shown that PI3K/AKT signaling activity enhanced by CTLA-4 plays a key role in maintaining the balance of T-cell survival, anergy, and apoptosis and in maintaining long-term immune tolerance (52). However, after combining with CD80/CD86, CTLA-4 recruits SHIP2 and PP2A to the membrane, the SHIP2 inhibits TCR signaling by dephosphorylation of CD3, as well as restrains PI3K/AKT signaling via dephosphorylation of PI3K, while PP2A directly dephosphorylates AKT to reduce its activity. The results indicate that the inhibitory activity of CTLA-4 depends on both PP2A and SHIP2, which coordinate the lower activity of NF-κB, mTOR, Bcl-xl, and the production of IL-2 in T cells (62), especially the reduced-AKT activity, which has been demonstrated to be required for the suppressive function of CD4^+^CD25^+^Foxp3^+^ regulatory T cells (63), and the differentiation and proliferation of natural Treg cells in the thymus are antagonized by PI3K/AKT signals via preventing FOXO factors translocating into the nucleus (50) (**Figure 2**). On the other hand, the study also reported that SHIP2 enhanced PI3K activity and was required for PDGF and IGF-induced AKT phosphorylation in mouse fibroblast (60). The effects of CTLA-4 on PI3K/AKT signaling are still debatable. The different results might come from different experimental conditions, cells, organs, and diseases, but nevertheless, the CTLA-4/PI3K/PKB pathway plays an important role in immune regulation, especially in the function of Treg cells.

## 4 The function of CTLA-4 on immunity

CTLA-4 inhibits immune responses by mediating various immune-related signaling pathways. It competes with B7 and CD28 via SYP, dephosphorylates p52^SHC^ in the CD3/p52^SHC^/GRB2/SOS complex to inhibit TCR signaling, and regulates TCR and the PI3K/AKT pathway via SHIP2 and PP2. Thus, CTLA-4 regulates T-cell signaling, modulates T-cell activity, maintains the balance of immunity, and protects the body from autoimmune diseases by downregulating the transcription factor activity of Fos, Jun, c-Myc, AP-1, NF-AT, and NF-κB (49), from which it protects the body from autoimmune diseases (**Figure 2**) (64). The mounting evidence regarding CTLA-4 provides an insight into the molecular underpinnings of T cells in their functions of immune suppression and immunological tolerance.

The mice lacking the *CTLA-4* gene showed dysregulation of the T-cell immune response, which exhibited a dramatically increasing T-cell blast in the lymph nodes and spleens, and the mice died at 3 weeks of age (65). Mice with a specific deletion of CTLA-4 in Treg cells disrupted the suppressive activity of the cells, spontaneously developed lymphocyte proliferative disease, and increased CD80 and CD86 expression in DCs, suggesting that the deficiency of CTLA-4 in Treg cells may inhibit the immune function of DCs (5). Another study indicated that in the absence of CTLA-4 in mice, naïve CD4^+^ T cells spontaneously differentiated into T follicular helper cells (Tfh), the number of germinal centers increased, and high levels of cytokines (IL-2, IFN-γ, IL-4, and GM-CSF) were produced (66). Similar results appeared in the blockage of CTLA-4 with antibodies, which further confirms that Tfh differentiation is regulated by CTLA-4 (67). However, Paterson et al.’s study showed that CTLA-4-depleted Treg cells still had suppressive activity and were sufficient to protect mice from EAE, as well as upregulate the immune regulators IL-10, LAG-3, and PD-1 expression (68). The patients with the CTLA-4 mutation had dysfunction of FoxP3^+^ Treg cells, hyper-proliferation of lymphocytes, and activated effector T cells, which resulted in a large number of lymphocytes in the circulation and in lymphoid organs (69). Overall, the evidence has indicated that CTLA-4 plays an important role in controlling effector T-cell differentiation, proliferation, and apoptosis and endowing Treg cell with immune suppressive activity.

Patients with deficient CTLA-4 have lower B-cell death and BCR-induced proliferation (70). However, the study also showed that the patients with CTLA-4 mutations had lower levels of circulating B cells and were associated with hypogammaglobulinemia and lymphopenia (69, 71). In mice, CTLA-4 deficiency produced a much higher frequency of activated B cells and an increased amount of immunoglobulin in their serum. CTLA-4 deletion in both Tfh and Tfr increased B-cell responses, whereas CTLA-4 deletion in Tfr alone increased antigen-specific antibody production (72). It has been demonstrated that CTLA-4 controls the activity of follicular helper T cells (Tfh) and downregulates co-stimulatory molecules of B cells, hence suppressing B-cell activation and antibody production (72).

CTLA-4 expression was detected on activated natural killer (NK) cells, but the function of CTLA-4 in NK cells is under investigation. The patients with CTLA-4 haploidy significantly reduced degranulation activity and production of IFN-γ in NK cells (73). The patients with CTLA-4 deficiency exhibited a reduced number of NK cells and impaired the function of NK cells, including cytotoxicity and inflammatory cytokine generation; the upregulated expression of CTLA-4 in activated NK cells provided an inhibitory signal for controlling NK cell activity and cytokine generation, which were confirmed by studying CD28 or CTLA-4 gene knockout mice and showing that NK cell IFN-γ production was negatively correlated with the level of CTLA-4 and positively correlated with the level of CD28 (74).

Research has shown that CTLA-4’s extracellular domain is sufficient to exert its inhibitory effect on T cells. Recent studies have demonstrated that CTLA-4 may decrease T-cell immunity even in the absence of the whole extracellular domain and has a similar ability to inhibit T-cell activation and cytokine expression *in vitro* (75). Overexpression of the cytoplasmic domain of CTLA-4 (cdCTLA 4) promoted naive T-cell preferential differentiation into Foxp3^+^ T cells in Th17 differentiation conditions via the reduction of MAPK phosphorylation and increased nuclear localization of Smad2/3 (76). Contemporary studies also indicated that cdCTLA-4 raised the number of follicular regulatory T (Tfr) cells and lowered the number of follicular helper T (Tfh) cells and germinal center (GC) B cells in draining lymph nodes (77). It implies that not only the extracellular domain, but also the cytoplasmic domain of CTLA-4 also has immunoinhibiting activity.

## 5 CTLA-4: a key regulator for autoimmunity

To maintain the body’s immune homoeostasis and silence of autoimmunity, the immune system needs to be tightly regulated, especially the activity of T cells because they play an important role in autoimmune regulation (31). After TCRs receive the first activated signal, the binding of CD28 with B7 on activated APCs enhances TCR signaling and prevents non-responsiveness or anergy of T cells. Following the activation of T cells, CTLA-4 is motivated to move to the cell membrane and compete with CD28 to bind to CD80/86 on activated APCs. The signal of CTLA-4, directly and via DCs, inhibits T-cell activation, proliferation, and cytokine production, thus downregulating T-cell activity (2, 26).

Research has demonstrated that CTLA-4 is a critical negative regulator of autoimmune diseases (78). Polymorphisms in the CTLA-4 locus, such as CTLA-4 + 49 G/A, CT60, −1661A/G, and many more, have long been associated with autoimmunity (79–81). The heterozygous mutations of CTLA-4 in T cells have been reported to be linked to a variety of autoimmune diseases (65, 82). The animal deletion of CTLA-4 leads to unregulated proliferation and activation of CD4^+^T cells (83, 84). In mice, the CTLA-4 deficiency promoted the proliferation of T cells that infiltrated into targeted tissues and caused organ damage, which suggests that CTLA-4 dysfunction can induce activation of self-antigen-specific T cells (85). The functional integrity of the CD28 molecule was necessary for CTLA-4 knockout mice to cause autoimmune diseases, implying that CTLA-4 suppresses the autoimmunity caused by the CD28 signaling pathway (86). Taken together, dysfunctions of CTLA-4 in T cells can cause a breakdown of immunological self-tolerance and result in susceptibility to autoimmune diseases.

Deletion of CTLA-4 in B-1a cells led to higher production of autoantibodies, increased the number of Tfh cells and germinal centers, and promoted cell differentiation into APCs and greater self-replenishment in the mice, which caused disruption of immune homeostasis, loss of immune tolerance, and the development of autoimmune disease in the late life of the mice (87). *In vitro* studies reported that B cells isolated from healthy donors treated with CTLA-4Ig, a fusion protein of the extracellular domain of CTLA-4 and IgG1, inhibited *Staphylococcus aureus*-induced CD80/CD86 expression on B cells, especially on the surface of the cell membrane, and TNF-α and IL-6 secretion from B cells (88). Besides lymphocyte inactivation, CTLA-4Ig can also inhibit the differentiation of osteoclasts and therefore regulate osteogenesis, suggesting that CTLA-4Ig may have the function of preventing bone destruction in rheumatoid arthritis (RA) (89).

## 6 CTLA-4: in the development of autoimmune diseases

The CTLA-4 gene has previously been shown to be associated with RA, systemic lupus erythematosus (SLE), multiple sclerosis (MS), type 1 diabetes (T1D), and myasthenia gravis (MG). Patients with autoimmune diseases (e.g., RA, SLE, MS, and T1D) have lower levels of CTLA-4 mRNA and protein in their PBMC, spleen, and lymph nodes than healthy subjects (**Table 1**).

**Table 1:** Expression status of CTLA-4 in different autoimmune diseases.

Disease	Expression Status	References
Rheumatoid arthritis (RA)	PBMC, spleen, lymph node ↓	(90, 91)
Systemic lupus erythematosus (SLE)	PBMC ↓	(92–94)
Multiple sclerosis (MS)	PBMC, spleen, lymph node ↓	(95, 96)
Type 1 diabetes (T1D)	Spleen and thymus cell↓	(97, 98)
Autoimmune thyroid disease (AITD)	Spleen and lymph node↓	(99–101)
Myasthenia gravis (MG)	PBMC↓	(102)

↓ denotes the downregulation or lower expression.

### 6.1 CTLA-4 and rheumatoid arthritis

RA is an autoimmune disease that is characterized by severe inflammation, hyperplasia of synovial lining cells, infiltration of mononuclear cells, and destruction of the articular joint. In addition, autoantibodies such as rheumatic factor (RF) and anti-citrullinated protein antibodies (ACPA) are also present in RA (103, 104).

**Figure 3:**
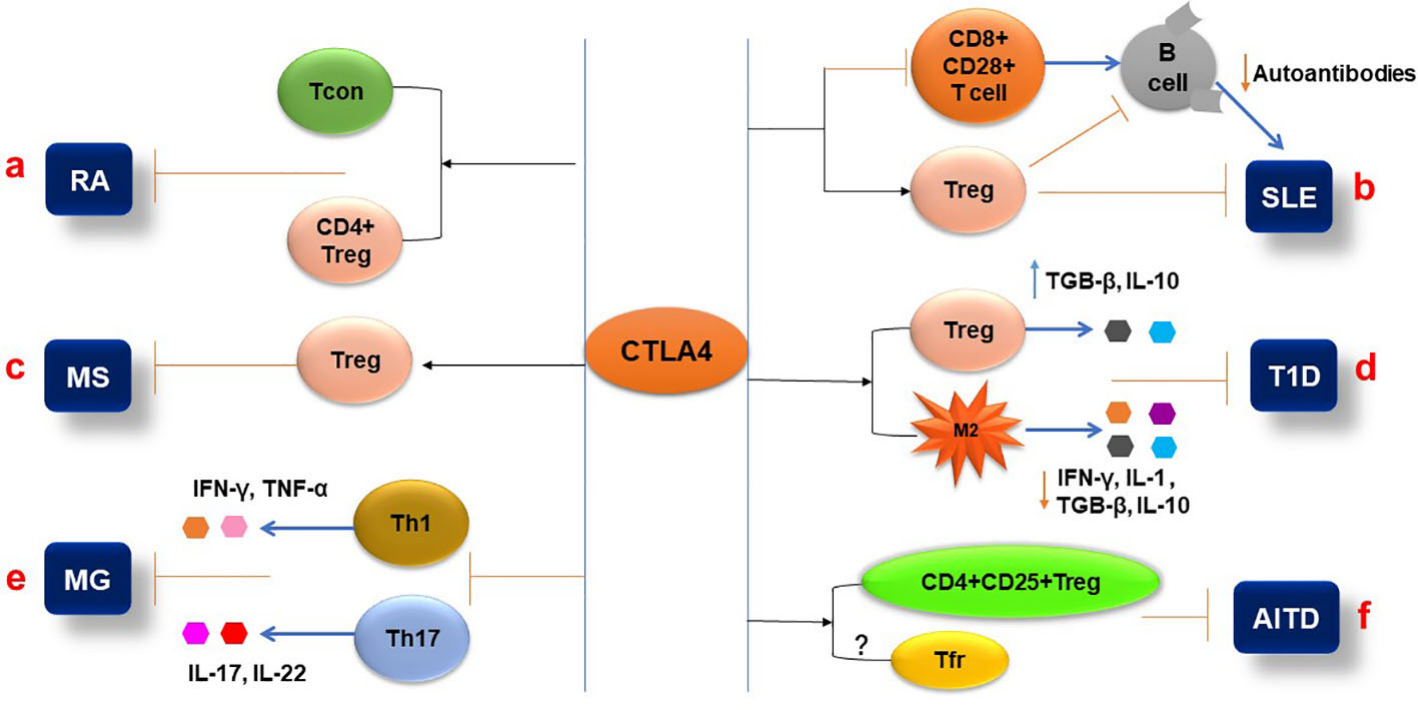
Pathophysiological aspects of autoimmune diseases are influenced by CTLA-4. **(A)** Expression of CTLA-4 in the T conventional and T regulatory cells (CD^+^FoxP3^+^) can inhibit the pathogenesis of RA. **(B)** CTLA-4 expression attenuates the Treg cell’s immune suppressive function and inhibits the CD8+ CD28+ T-cell functions that affect B-cell production of autoantibodies during SLE pathogenesis. **(C)** The CTLA4 signaling molecule can activate Treg cells and reduce MS activity. **(D)** CTLA-4 differentiates the CD4^+^CD25^+^ Treg cells and inhibits T1D pathogenesis by increasing the secretion of IL-10 and TGF-β. Additionally, CTLA-4 interaction with macrophages reduces pro-inflammatory cytokine (IL-1 and IFN-γ) secretion and inhibits T1D. **(E)** In MG, CTLA-4 expression decreases the frequency of Th1 and Th17 cells and their cytokine production. **(F)** In AITD, the CTLA-4 plays an inhibitory role by activating the CD4^+^CD25^+^ Treg cells and their immunosuppressive functions.

CTLA-4 is a molecule that participates in the regulation of T-cell activity during autoimmune response, and multiple CTLA-4 single-nucleotide polymorphisms (SNPs) have been demonstrated to be closely associated with RA. Meta-analysis showed that polymorphisms of CTLA-4 (rs3087243, rs5742909, rs231775, and CTLA-4 + 49A/G) were significantly associated with the risk of RA (105–107). Klocke et al. (108) revealed that the expression of CTLA-4 by FoxP3^+^ regulatory T (Treg) cells attenuated the activity of disease and prevented tissue damage. They also found that overexpression of CTLA-4 in conventional T (Tcon) cells inhibited collagen-induced arthritis (CIA) by repressing the activity of T cells (**Figure 3A**). RA patients in the quiescent stage of the disease have lower levels of sCTLA-4 than the patients in the activating stage (90). CTLA-4 deficiency in Treg cells from RA patients significantly reduces their immune suppressing activity (91). Compared with the normal group, CTLA-4 expression in CD4+ Foxp3+ cells in rheumatoid arthritis patients was reduced, which was associated with an increased rate of CTLA-4 internalization; artificially driving CTLA-4 to the T cell surface with PMA restored the suppressive function of the cells, but this restoration can be reversed by CTLA-4 inhibition (91). The methylation of the CTLA-4 promotor’s DNA at the NF-AT binding site resulted in insufficient CTLA-4 expression in RA patients’ Treg cells, which, in turn, leads to the failure of the expression and activation of the tryptophan degrading enzyme indoleamine 2,3-dioxygenase (IDO); as a consequence, the Treg cells were unable to activate the kynurenine pathway, which exacerbates the development of the RA (109). RA clinical trials showed that the CTLA-4-Ig fusion protein Abatacept can reduce synovial inflammation and pathology by selectively modulating CD28, CD80, and CD86 co-stimulation signals in T cells (110, 111), which further confirmed that the functions of CTLA-4 in immune regulation, especially in Treg cells, play a very important role in controlling the onset and progression of RA (**Figure 3A**).

### 6.2 CTLA-4 and systemic lupus erythematosus

SLE is a systemic autoimmune disease resulting from autoimmune responses against nuclear autoantigens. In SLE, the body’s immune system attacks various self-tissues and damages a number of organs, including the kidney, brain, skin, and joints. Although the exact pathogeny of SLE is not clear, both genetic and environmental factors might be associated. Hyperactive B cells and the production of autoantibodies are common in SLE (112). The list of candidate genes related to SLE pathology is lengthy, especially MHC and CTLA-4. Several meta-analyses have reported that the CTLA-4 exon-1 + 49 (A/G) polymorphism is responsible for the development of SLE, especially in Asians (113, 114). Three other meta-analyses describe that CTLA-4 promoter -1722T/C, CT60A/G, and -318C/T polymorphisms also confer risk to SLE development in Asians and Iranians (115–117).

CTLA-4 is a concern for SLE-related studies due to its inhibitory role in immune responses and control of hyperactive T and B cells, the mechanism of which may be through the interaction between auto-reactive B cells and CD4^+^ T cells and the CD28/CD80-86/CTLA-4 axis. Simulating the effect of CTLA-4 competing with CD28 for B7, anti-CD28 was used to block CD28 signaling in NZB/NZW mice, which prevented lupus nephritis development, prolonged animal survival, reduced production of against double strand DNA (dsDNA) autoantibodies, and increased expression of IDO, receptor programmed cell-death-1 (PD-1), and ligand programmed death ligand-1 (PDL-1) (118). The CD8^+^CD28^+^ T cell subset of PBMCs from patients with active SLE expressed a lower level of CTLA-4, suggesting that the CD8^+^CD28^+^ T cells with higher activity may result from a lower expression of CTLA-4, which leads to the development of SLE (92) (**Figure 3B**). Further research showed that compared to the control group, the CTLA-4 levels of CD4^+^CD25^+^T cells and CD4+CD25^+^FoxP3^+^Treg cells isolated from the patients with SLE were significantly reduced and were negatively correlated with the SLE disease activity index and severity (93). Consistent with this result, a study indicated that the frequency of CTLA-4^+^ Treg and CD28^+^ Treg cells in peripheral blood mononuclear cells (PBMCs) of patients with SLE was also lower than that of healthy individuals (94). The low expression of CTLA-4 in patients with SLE leads to a decrease in the immune regulatory ability of Treg cells, an increase in autoimmune activity, and the deterioration of SLE. CTLA-4 has been reported to be essential for reducing Treg-inhibited effector T-cell proliferation, decreasing inflammatory cytokine release (119), and maturing of inducible Treg (iTreg) cells (120) (**Figure 3B**). Interestingly, another study showed a higher level of CTLA-4 in FOXP3^-^ T cells from patients with SLE compared to other autoimmune diseases and healthy controls, but their study indicated that the FOXP3^-^ T cells in SLE patients were unable to control activation and proliferation of effector T cells (121). The studies demonstrated that CD8^+^ CD28^+^ T cells secrete IL-2 and IFN-γ and stimulate B-cell proliferation and function to produce antibodies (122, 123), and that mechanism directly modulates SLE development. Further study highlighted that CD8^+^ T cells can modulate various cytokines, including CTLA-4, and play a role in the pathogenesis of autoimmune diseases such as SLE, MS, and T1D (124).

Substantial evidence has demonstrated that genetic changes, decreased expression, and function abnormalities of CTLA-4 increase the risk of developing SLE and contribute to the onset and progression of SLE (121, 125). The drugs targeting CTLA-4 are being investigated as potential treatments for SLE.

### 6.3 CTLA-4 and multiple sclerosis

MS is a chronic autoimmune disease that affects the brain and central nervous system (CNS). In MS, the body’s immune cells usually attack the myelin sheath that covers nerve fibers and disrupts the connection between the brain and other parts of the body. In addition, auto-reactive T cells play a vital role in initiating the self-reactive immune response (126). The etiology of MS is unknown, but some studies suggest that either a viral infection or a direct autoimmune process is responsible for this disease. Currently, researchers are focusing on the role of inhibitory receptors in T cells, especially CTLA-4 and PD-1. It has been established that CTLA-4 is associated with MS genetically. The polymorphism of CTLA-4 has been reported to be associated with MS pathology. The G allele in the rs231775, A>G (+49 A>G) polymorphism of CTLA-4 contributes to the reduction of auto-reactive T-cell activation and leads to the development of MS (127). In contrast, two meta-analyses reported opposite results about CTLA-4 polymorphism and MS (128, 129).

Several studies have reported defective expression of various inhibitory receptors, such as CTLA-4, PD-1, and TIM-3, in MS patients. Freshly isolated PBMCs from MS patients showed lower levels of CTLA-4, PD-1, and TIM-3 than those from healthy people (95). Consistent with this finding, compared to healthy controls, lower surface and higher intracellular expression of CTLA-4 in CD4^+^CD25^+^ T cells were found in MS patients, and these were correlated with the levels of FoxP3 mRNA (96).

In experimental autoimmune encephalomyelitis (EAE) in mice, blocking CD80/CD86 molecules with CTLA-4Ig increased disease score with increased production of interleukin-17 (IL-17) and interferon-γ (IFN-γ). On the other hand, the CTLA-4Ig-treated EAE mouse model drastically reduced the number of CD4^+^FoxP3^+^ Treg cells and level of CTLA-4 compared to the untreated EAE mouse model (130). A study reported that CTLA-4 signaling peptide can induce Treg cells and inhibit the activity of MS (131) (**Figure 3C**). Furthermore, a cysteine-containing cell-penetrating peptide (AP)-conjugated CTLA-4 cytoplasmic domain (AP-ctCTLA-4) peptide attenuated the activity of EAE by inhibiting IL-17A expression and reducing the number of pathogenic IL-17A^+^GM-CSF^+^ CD4 T cells (132). The results suggest that CTLA-4 is a target for the treatment of MS.

### 6.4 CTLA-4 and type 1 diabetes

T1D, once known as juvenile diabetes, is a chronic immune disorder in which the patients’ immune system destroys insulin-making pancreatic β cells, which are mediated by T cells, pro-inflammatory macrophages, and DCs (133). The underlying mechanism that causes T1D onset and progression is unknown. Genetic predisposition and environmental factors may play vital roles in this pathogenesis. A recent study reported that various genes have been associated with the pathogenesis of T1D. To date, research that shows that specific allele combinations like DRB1* and DQB1* in HLA are associated with T1D has been reported (134). Like other genes, the association between T1D and the polymorphism of CTLA-4 has been studied in several meta-analyses. One study described that the polymorphism of CTLA-4 (+49 A/G) is strongly associated with T1D in the south Indian population (134). A homogeneous combination, such as CTLA-4 + 49 GG/AA genotypes combined with HLA high risk alleles, confers a risk of T1D development than a heterogeneous gene combination. Further supporting this evidence is another meta-analysis that reported that the polymorphism of CTLA-4 + 49 G/A (rs231775) is associated with autoimmune diseases such as T1D, rheumatoid arthritis, and SLE in Asian and Caucasian populations (135). One study in Egypt revealed that the frequency of CTLA-4 polymorphism (+49 A/G) significantly increased in the T1D group than that in the control group, particularly in younger patients and female patients (136).

Regulatory T cells, including natural regulatory T cells (nTreg) and peripheral-induced regulatory T cells (iTreg), are coordinated to maintain immune homeostasis. A reduction in frequency and/or function of Treg cells is one of the main reasons for breaking the immune tolerance of the immune system to β cells and causing T1D (137, 138). The study showed that CTLA-4 plays a key role in controlling Treg cell-mediated immunological tolerance (5). According to a recent study, blocking CTLA-4 in non-obese diabetic (NOD) mice at 10 days of age induced mice to develop T1D more quickly than the control group. This result showed that CTLA-4 is essential for Treg cell differentiation and function in the NOD model (139). Wang and his colleagues described that the expression and membrane trafficking of CTLA-4 were significantly higher in Treg cells than in conventional T cells isolated from the pancreases of the DO11×RIP-mOVA diabetic mouse model, suggesting that the Treg cell CTLA-4 plays an important role in the regulation of diabetic immunity (97). In diabetic patients, Treg cells expressed lower CTLA-4 compared to the control group (98). The patients with melanoma were treated with anti-PD-1 or anti-CTLA-4, which increased part of the patients’ glycemia levels and caused T1D and type 2 diabetes (T2D) during the immunotherapy period. Based on a high level of C-reactive protein (CRP), they believe that the pathogeny of diabetes may be insulin resistance caused by inflammation (140) (**Figure 3D**).

The obesity-induced diabetic mouse model treated with CTLA-4Ig dramatically improved insulin sensitivity by promoting macrophage differentiation into M2 macrophages, which increased anti-inflammatory cytokine (IL-10 and TGF-β) and reduced proinflammatory cytokine (IL-1 and IFN-γ) production (141) (**Figure D**). One mechanism by which ethyl pyruvate (EP) reduced the incidences of streptozotocin-induced T1D was by increasing the level of CTLA-4 in CD4+CD25^high^FoxP3^+^ Treg cells, which also increased the expression of TGF-β and IL-10 (142) (**Figure 3D**). On the other hand, inhibition of CTLA-4 accelerated the development of T1D, such as miR-487a-3p, which promoted T1D development by suppressing CTLA-4 and FOXO3 through binding to their 3′UTR regions (143).

### 6.5 CTLA-4 and myasthenia gravis disease

MG is defined as a long-term neuromuscular disease characterized by weakness and rapid fatigue of skeletal muscle. It is caused by interrupted communication between nerve and muscle cells at the neuromuscular junction (NMJ) (144). The destruction of the neuromuscular junction is due to the production a number of autoantibodies against acetylcholine receptor (AChR), muscle-specific kinase (MuSK), and LRP4 (145). Most of the autoantibodies (IgG1 and IgG3) are anti-AChR that inhibit the binding and degradation of muscle acetylcholine receptors. MuSK is a receptor tyrosine kinase that is activated by agrin and essential for NMJ formation. LRP4 is an agrin receptor that is required for agrin-induced activation of MuSK and AChR clustering. The effect of anti-MuSK is to block NMJ formation, and anti-LRP4 antibody aims to interfere with the activation of MuSK and AChR clustering. Together, these autoantibodies lead to the damage of NMJ formation, destruction of NMJ, and disruption of the signal transduction of NMJ (144, 145).

People who have rs733618, rs231775, and rs3087243*G polymorphisms in the CTLA-4 gene have increased susceptibility to MG (146). Functionally abnormal Treg cells were found in MG patients with low levels of CTLA-4 and CD25 (102). The hypermethylation at −658 and −793 CpGs of the CTLA-4 promoter has been indicated to be associated with MG by decreasing the frequency of Treg cells and CTLA-4^+^ Treg cells (147). The level of methylation was positively correlated with the level of anti-acetylcholine receptor (AChR) antibodies in MG patients. Th1 and Th17 CD4+ T cells and their cytokines IFN-γ and IL-17 showed that they drive anti-AChR and MuSK antibody production via B cells (148). Anti-CTLA-4 antibody increased the frequency of Th1 and Th17 cells and their cytokines IL-2, IFN-γ, and IL-17, respectively (149, 150). These imply that the abnormal CTLA-4 in Treg cells is associated with the generation of the antibodies (**Figure 3E**).

### 6.6 CTLA-4 and autoimmune thyroid disease

Autoimmune thyroid disease (AITD) is characterized by a loss of immunological tolerance for the thyroid tissue and damaged thyroid function. AITD includes Graves’ disease (GD) and Hashimoto’s thyroiditis (HT). In AITD, lymphocytic infiltration causes tissue damage and changes the function of the thyroid gland. It was shown that autoantibodies or autoreactive T cells are responsible for thyroid tissue injury or inflammation (151). The environmental factors and genetic associations are described in the multifactorial etiology of AITD.

Research has demonstrated a close relationship between CTLA-4 and AITD, including GD and HT (152). The studies demonstrated that the polymorphisms of CTLA-4 such as +49A/G and CT60, but not the -318C/T, were found to have a significant correlation with the risk of HT (153–155). According to recent studies, the +49A/G CTLA-4 polymorphism has been demonstrated to link to Down syndrome disorders in HT patients (156), correlate with antithyroid antibody production in children with HT (157), and increase susceptibility and relapse of GD (158, 159). Additionally, the results of using sodium iodide to induce AITD in NOD-H2h4 mice and of the treatment or non-treatment with anti-CTLA-4 antibody indicated that the amount of mononuclear cell infiltration in the thyroid as well as CD4^+^ effector T cells in the spleen and the level of thyroglobulin were significantly higher in the anti-CTLA-4-treated group than those in the control group (99). The study showed that CTLA-4 plays a key role in the immune suppressive function of naturally occurring CD4^+^CD25^+^ T (nTreg) cells that is essential for inducing immune tolerance in murine experimental Hashimoto’s thyroiditis (EHT) (100) **(** **Figure 3F** **)**. Follicular T-helper (Tfh) cells promote the pathogenesis of AITD. The cell surface expression of CTLA-4 in T cells was higher in HT patients than in the control group; after phytohemagglutinin (PHA) stimulation for 48 h, the number of CD4 T cells expressing CTLA-4 increased in both HT patients and controls, but CTLA-4 expressed on the cell surface increased only in HT patients (101). Follicular helper T cells (Tfh) play a crucial role in the development and maintenance of lymphomatic germinal centers and provide key signals for germinal B cells to undergo somatic hypermutation, selection, and high-affinity maturation, which results in germinal B cells differentiating into plasma cells that produce high-affinity antibodies. Tfh cells have been found to facilitate the development of autoantibodies that target self-antigens in autoimmune disorders like HT (160). In patients with HT, Tfr cells are thought to inhibit the production of autoantibodies by suppressing the function of Tfh cells. This is achieved by producing anti-inflammatory cytokines such as IL-10 and TGF-β, which inhibit the proliferation and function of Tfh cells (161). In patients with GD, the number of circulated Tfh negatively correlated with serum concentrations of TSH receptor antibodies (162) (**Figure 3F**). Studies have demonstrated that expression of CTLA-4 in Tfr cells is essential for the immune suppressive activity of the cells, but paradoxically, Zhao et al. showed in their study that the percentage of CTLA-4 on Tfr cells was significantly reduced in patients with HT (161). APCs interact with CTLA-4 on T cells to suppress the activation and proliferation of the cells and promote the development of Treg cells (163). Therefore, it is thought that the interaction between Tfr cell CTLA-4 and APC CD28 is crucial for maintaining immunological tolerance and preventing the emergence of HT and GD. Tfh cells and B cells may become uncontrollably activated because of dysfunction or a lack of Tfr cells or CTLA-4, which leads to the production of autoantibodies and the autoimmune destruction of thyroid tissue (164, 165). This highlights the importance of Tfr cells and CTLA-4 in maintaining immune homeostasis and preventing AITD.

## 7 Prospect of CTLA-4 as autoimmune disease therapeutics

Although there is no reported association between CTLA-4 and sex, it is known that conditions such as SLE, AITD, RA, and MS are more prevalent in women than men. Genetic analysis of CTLA-4 (**Table 2**) has reported that co-stimulatory pathways are closely related to ADs. The study illustrated that deletion of CTLA-4 in adult mice leads to autoimmune disease (85). Nowadays, researchers have shown the CTLA-4 immunoglobulin (CTLA-4Ig) fusion protein has treatment effects for autoimmune diseases (**Table 3**). The first soluble CTLA-4Ig antibody, Abatacept, showed promising effects in RA clinical trials (166). The following improved variants of CTLA-4Ig, such as Belatacept, XPro95, and MEDI5256, especially MEDI5256, not only have 128-fold greater binding affinity to CD80 and CD86 than Abatacept but also have higher stability and longer pharmacokinetics (167, 168).

**Table 2:** The list of *polymorphisms* associated with ADs.

Disease	Polymorphisms	References
Rheumatoid arthritis (RA)	rs3087243, rs5742909, rs231775, and +49A/G	(105–107)
Systemic lupus erythematosus (SLE)	+49 (A/G), −1722T/C, CT60A/G, and −318C/T	(113–117)
Multiple sclerosis (MS)	rs231775 and +49 A/G	(127)
Type 1 diabetes (T1D)	+49 A/G	(134, 135)
Myasthenia gravis (MG)	rs733618, rs231775, and rs3087243	(146)
Autoimmune thyroid disease (AITD)	+49A/G and CT60	(153–155)

**Table 3:** Role of anti-CTLA-4/CTLA-4Ig in different autoimmune diseases.

Disease	Role of anti-CTLA4/CTLA-4Ig	References
Rheumatoid arthritis (RA)	Enhance Treg cell activity and reduce B-cell activity	(108, 173)
Systemic lupus erythematosus (SLE)	Inhibits to produce hyperactive T cells and promotes Treg cell induction	(119, 120)
Multiple sclerosis (MS)	Reduce the CD4+FoxP3+ Treg cell expression and CTLA-4 expression in the EAE mouse model	(130)
Type 1 diabetes (T1D)	Improve insulin sensitivity and macrophage differentiation and reduce proinflammatory cytokine production	(139)

Recently, abatacept (CTLA-4Ig) has become a new approach for RA immunotherapy (169). Treatment with abatacept (CTLA-4Ig) for rheumatoid arthritis considerably decreased disease severity by preventing T-cell proliferation, lowering the level of pro-inflammatory cytokines, and perhaps decreasing the quantity of autoreactive T cells (170–172). The study reported that CTLA-4Ig treatment reduces B-cell activity and also enhances the inhibitory capacity of Treg cells in RA patients (173, 174). The experiments suggested that CTLA-4Ig may govern humoral responses by interfering with the interaction between CD28 of T cells and CD80/CD86 of B cells, thus blocking the CD80/CD86 signal in B cells (88). Contradictorily, studies have shown that CTLA-4-Ig significantly increases the proportion of CD4^+^T and Treg cells by reducing the level of CD95 in the cells (175), but further analysis showed that Treg cell suppressive capacity and responsive T-cell proliferation ability were weakened in RA patients (176). There was a decrease in myelin basic protein proliferation and IFN-γ secretion in patients with MS treated with CTLA-4Ig (177). On the other hand, the study indicated that the patients who received anti-CTLA-4 antibody (ipilimumab) treatment showed clinical episodes of MS, which further confirms that CTLA-4 is a treatment target for MS (178). In individuals with T1D, treatment with Abatacept had a favorable safety profile (179). During the 2-year period of taking Abatacept, T1D patients continued to slow β cell damage and functional decline as well as maintain a low level of HbA1c, and these effects persisted for at least a year after the antibody cessation (180). In patients with newly diagnosed T1D, if they had been treated with Abatacept, the decreased rate of C-peptide significantly slowed down (181).

So far, AITD (182), SLE (183), diffuse cutaneous systemic sclerosis (184), MG (185), celiac disease (186), and allergic asthma (187) do not have the results of clinical trials for CTLA-4-Ig, but the clinical study indicated that a higher level of sCTLA-4 was found in the serum of the above autoimmune disease patients. In addition, studies showed that sCTLA-4 reduced the levels of proinflammatory cytokines such as IFN-γ, IL-2, IL-7, and IL-13 and increased the production of anti-inflammatory cytokines TGF-β and IL-10. These results imply that CTLA-4 can be a potential target for treatment of these autoimmune diseases.

## 8 Conclusion

Tissue damage or organ malfunction can result from the body’s immune system attacking its own self-antigens. Based on the T cell being located at the center of immune regulation, the functions and activities of Treg cells play a prominent role in the maintenance of body immune homeostasis. CTLA-4 and CD28 are two critical molecules that share a common ligand, CD80/CD86, on APCs required for T-cell regulation. CTLA-4 has the opposite effect of CD28 on T-cell immunity through competitive binding of CD80/CD86, thus blocking the second activating signal for T-cell activation and resulting in anergy and clonal tolerance of T cells, which also block DC differentiation and become immune tolerogenic cells. More and more data have suggested that CTLA-4 is crucial in the onset and progression of autoimmune diseases, and this is confirmed by the effects of CTLA-4Ig in the treatment of autoimmune diseases. However, it is worth noting that the mechanism by which CTLA-4 regulates the immunity of Treg cells, B cells, NK cells, DC, and macrophages needs to be further studied, as well as the effects of this molecule on endothelial, epithelial, and fibroblast immunoregulation, and its roles in the treatment of various autoimmune diseases. It is reasonable to believe that through the study of the CTLA-4 immune regulation signaling pathways and effects on various autoimmune diseases, there will be benefits for the treatment of autoimmune diseases.

## Author contributions

MH and YM reviewed the literature, generated figures, and wrote the paper. ZHY, YX, and JD reviewed the literature, revised the paper, and offered feedback on the draft manuscript. JYH, JJH, and LZ reviewed the literature. ZZY and ZH designed the concept of the work, reviewed the literature, and wrote and edited the paper. All authors contributed to the article and approved the submitted version.

## Glossary

**Table:**

ADs	autoimmune diseases
CTLA-4	cytotoxic T-lymphocyte antigen 4
APCs	antigen-presenting cells
MHC	major histocompatibility complex
flCTLA-4	full-length cytotoxic T-lymphocyte antigen 4 mRNA
sCTLA-4	soluble cytotoxic T-lymphocyte antigen 4
liCTLA-4	ligand-independent cytotoxic T-lymphocyte antigen 4
NF-AT	nuclear factor of activated T cells
cAMP	cyclic adenosine monophosphate
TGN	trans-Golgi network
GTPases	guanosine triphosphatases
ARF-1	adenosine diphosphate ribosylation factor-1
PLD	phospholipase D
CAP-1	clathrin adaptor protein-1
CAP-2	clathrin adaptor protein-2
IL-2	interleukin 2
TCR	T-cell antigen receptor
Bcl-Xl	apoptosis regulator Bcl-X
LAT	linker for activation of T cells
NF-κB	transcription factor nuclear factor B
Treg	regulatory T cell
DCs	dendritic cells
GRB2	growth factor receptor bound protein
SOS	Son-of-Sevenless
SYP	tyrosine phosphatase synaptophysin
CXCR4	C-X-C chemokine receptor type 4
PIP3	phosphatidylinositol (3
4	5)-trisphosphates
PH	pleckstrin homology
PDK1	PH domain kinase 1
mTORC2	rapamycin complex 2
PP2A	serine/threonine phosphatase PP2A
SHIP2	tyrosine phosphatase SHIP2
BAD	BL2-associated agonist of cell death
cdCTLA 4	cytoplasmic domain of CTLA-4
Tfr	follicular regulatory T
Tfh	follicular helper T
GC	germinal center
RA	rheumatoid arthritis
RF	rheumatic factor
ACPA	anti-citrullinated protein antibodies
Treg	regulatory T cell
Tcon	conventional T
IDO	indoleamine 2, 3-dioxygenase
SLE	lupus erythematosus
MS	multiple sclerosis
CNS	central nervous system
IFN-γ	interferon-γ
AP-	cell-penetrating peptide (AP)-conjugated
ctCTLA-4	CTLA-4 cytoplasmic domain
T1D	type 1 diabetes
NOD	non-obese diabetes
CRP	C-reactive protein
AITD	autoimmune thyroid disease
HT	Hashimoto's thyroiditis
EHT	experimental Hashimoto's thyroiditis
MG	myasthenia gravis
NMJ	neuromuscular junction
AChR	acetylcholine receptor
MuSK	muscle-specific kinase
AChR	anti-acetylcholine receptor
CTLA-4Ig	CTLA-4 immunoglobulin
